# Unraveling the Potential of *Chondrosia reniformis* Collagen for Tissue Engineering Scaffolds, with Particular Insights into Chondrogenic Differentiation

**DOI:** 10.1021/acs.biomac.4c01793

**Published:** 2026-01-08

**Authors:** Miguel S. Rocha, Ana C. Carvalho, Catarina F. Marques, Filipa Carneiro, Rita O. Sousa, Eva Martins, Eleonora Tassara, Rui L. Reis, Tiago H. Silva

**Affiliations:** † 3B’s Research Group, I3Bs−Research Institute on Biomaterials, Biodegradables and Biomimetics, 56059 University of Minho, Headquarters of the European Institute of Excellence on Tissue Engineering and Regenerative Medicine, AvePark, Parque de Ciência e Tecnologia, Rua Ave 1, 4805-694 Barco/Guimarães, Portugal; ‡ ICVS/3B’s−PT Government Associate Laboratory, 4806-909 Braga/Guimarães, Portugal; § Department of Earth, Environment and Life Sciences (DISTAV), 9302 University of Genova, Via Pastore 3, 16132 Genova, Italy

## Abstract

Evaluating the biomedical potential of marine biopolymers is a promising strategy for their high-value application. This study investigated the ability of collagen derived from *Chondrosia reniformis* to support cell proliferation and chondrogenic differentiation, assessing its suitability for tissue regeneration. Collagen was isolated, preserving its fibrillar structure and glycosylation features, then cross-linked with EDC, genipin, or glutaraldehyde to produce freeze-dried scaffolds. The resulting structures were characterized in terms of physicochemical properties, morphology, degradation, rheology, and cytocompatibility. While all scaffolds showed comparable degradation and rheological behavior, genipin-cross-linked scaffolds exhibited larger pore sizes, whereas glutaraldehyde-cross-linked scaffolds showed higher water uptake. *In vitro* assays using ATDC5, BJ, and EA.hy926 cell lines demonstrated superior metabolic activity and proliferation on genipin-cross-linked scaffolds. Additionally, human adipose stem cells displayed early chondrogenic differentiation, evidenced by *SOX9*, *ACAN*, and *COMP* expression under basal conditions. These findings highlight the versatility of *C. reniformis* collagen for biomedical applications, particularly cartilage regeneration.

## Graphical abstract

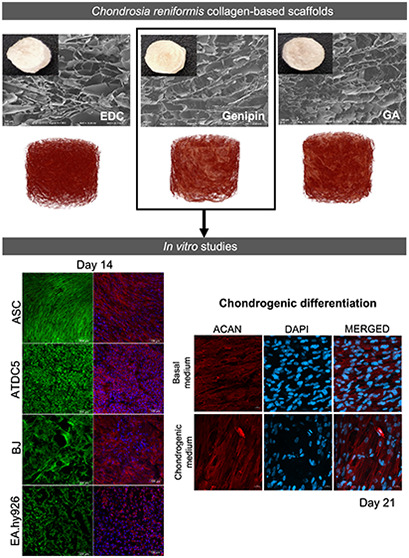

## 1 Introduction

A new paradigm is arising in Medicine to tackle loss of tissues due to trauma, disease, or wear, aiming at their regeneration instead of using grafts or artificial substitutes with limited outcomes. This Regenerative Medicine follows approaches as Tissue Engineering (TE), stem cell therapies, gene therapies, or combinations of these.^1–3^ TE continues to require innovative biomaterials presenting adequate mechanical properties, low toxicity and immunogenicity, while supporting (or, preferably, stimulating) cell proliferation and differentiation toward new tissue formation. Regarding stem cell therapy, present key issues include the development of scaffolds and/or factors that facilitate the recovery of stem cells from cryopreservation and support controlled differentiation, as well as determining the optimal number of stem cells required for a successful treatment.^4^

Many biopolymers are available for the production of biomaterials, among which collagen has proven to be effective for cell culture scaffolding, aiming at the regeneration of various tissues.^5,6^ While mammalian-origin collagen is still the primary industrial source for biomedical applications, marine-origin collagens have proven to be a suitable option.^7,8^ Although presenting clear advantages when compared with their mammalian counterparts, such as prevention of zoonosis (e.g., bovine spongiform encephalopathy, transmissible spongiform encephalopathy, and foot-and-mouth disease) and avoidance of religious and ethical concerns, marine collagens remain a largely unexploited resource with great potential to be sustainably explored.^9,10^ Furthermore, since human recombinant collagen production is hindered by low extraction yields and high costs, marine organisms become an even more compelling source in the growing demand for collagen.^11^

Marine collagens have already been successfully used in several TE approaches, ranging from skin to bone regeneration, demonstrating their high potential and wide applicability.^8,12–14^ Despite the main marine collagen sources currently being fish bycatch and processing industry byproducts, there are other sources available.^10^ Currently, collagen can be extracted from a wide variety of marine animals such as echinoderms, jellyfish, soft corals, and sponges,^8^ among which *Chondrosia reniformis* is presently one of the most promising and accessible alternatives. This marine sponge is mainly constituted by collagen and can be produced in integrated mariculture methods, enabling its sustainable and massive production.^15^ Marine sponge-derived collagen has already been investigated for a number of purposes, such as bone graft applications,^16^ drug delivery system,^17,18^ and even as a decellularized 3D structure for bone regeneration,^19^ yielding encouraging results. More specifically, *C. reniformis* collagen has recently been successfully employed in the development of biomedical applications aiming at skin regeneration.^20,21^ The membranes were produced employing a suspension of intact collagen fibrils and supported both fibroblast and keratinocyte cell cultures while improving fibronectin production, thus showing promise for wound healing applications.^20,21^ Nonetheless, *C. reniformis* collagen is still underexploited, thus possibly being suitable for additional TE strategies. In particular, cartilage tissue regeneration strategies are still limited to a few commercially available products, such as Chondro-Gide,^22^ although some bioactive substitutes have been proposed and developed over the past few years.^23^ Human native cartilage, being an avascular tissue, can have its functionality seriously compromised due to injuries and degenerative conditions, as generally damage is physiologically irreversible, thus requiring specific treatment.^24^ Additionally, as life expectancy grows, cartilage-related diseases rise accordingly, thus demanding the development of effective alternative treatments.

Scaffolding using exclusively collagen usually results in structures with inadequate mechanical properties and degradation rates, which should be tailored according to the desired application (as the tissue to regenerate). Thus, most commonly, collagen is either combined with other support materials or reinforced by employing cross-linkers. Cross-linking processes induce the formation of stable intra- and intermolecular chemical bonds, thus increasing collagen resistance to temperature and enzymatic degradation while enhancing its mechanical properties. There are different types of techniques available for collagen structure reinforcement, including physical, enzymatic, and chemical cross-linking.^25^ Distinct cross-linking methods present specific advantages, but may cause undesirable consequences, such as cytotoxic effects, and therefore a compromise must be achieved to find a biologically adequate solution.

In the present work, we hypothesized that *C. reniformi* *s* collagen can be used as a building block for the production of scaffolds envisaging the engineering of different tissues (namely, skin, cardiovascular, and cartilage) and, ultimately, to promote the chondrogenic differentiation of stem cells. To address this, *C. reniformis* collagen was chemically cross-linked with glutaraldehyde (GA), 1-ethyl-3-(3-(dimethylamino)­propyl)­carbodiimide hydrochloride (EDC), or genipin (frequently used collagen cross-linkers^25,26^ ) and freeze-dried to render scaffolds that were characterized regarding their morphological and mechanical properties, as well as swelling and degradation rates. The most promising scaffolds were further studied to unravel its versatility for the development of TE approaches, namely, regarding tissues such as skin, cardiovascular and cartilage, with particular focus in the latter, by evaluating their eventual cytotoxicity toward four cell types (ATDC5, BJ, EA.hy926 and human adipose stem cells (ASCs)) and their potential to promote ASC differentiation into chondrogenic lineage.

## 2 Materials and Methods

### 2.1 Materials

Frozen *C. reniformis* specimens were kindly provided by partners of Wageningen University & Research and transported in dry ice containers to the facilities of the University of Minho, Portugal, where they were stored at −20 °C until further use for collagen extraction. All reagents were purchased from Sigma-Aldrich (St. Louis, MO, USA), unless otherwise stated.

### 2.2 *C. reniformis* Collagen Isolation

Collagen isolation was performed as described in ref 27. All steps for collagen extraction were carried out at 4 °C. Marine sponge samples were thawed, and exogenous materials were removed. As the ectosome was reported to be more suitable for the development of biomaterials than the choanosome,^28^ it was isolated, cut into small pieces, and placed in disaggregating solution (50 mM Tris–HCl buffer, pH 7.4, 1 M NaCl, 50 mM EDTA, and 100 mM 2-mercaptoethanol) under stirring for 5 days. The resulting collagen suspension was extensively dialyzed against dH_2_O and then centrifuged for 10 min at 1200*g* (5810 R, Eppendorf, Hamburg, Germany) to remove cell debris and sand particles. A centrifugation for 30 min at 12,100*g* (5810 R) was performed to collect the collagen fibrils from the suspension, yielding collagen-containing pellets. To improve collagen’s purity, some of the polysaccharidic components coextracted with sponge collagen were partially removed by a treatment with an equal volume of 0.1 M NaOH for 6 h at room temperature (RT), followed by centrifugation at 12100 g for 30 min, with two further washing steps with PBS.^20,21,29^ Collagen pellets were freeze-dried and stored at RT until further use.

### 2.3 *C. reniformis* Collagen Characterization

#### 2.3.1 Fourier-Transform Infrared Spectroscopy in Attenuated Total Reflection Mode (FTIR-ATR)

The collagen infrared profile was recorded using Fourier-transform infrared spectroscopy (FTIR) in attenuated total reflectance (ATR) mode employing freeze-dried material. Measurements were conducted with an IR-Prestige-21 spectrophotometer (Shimadzu Scientific Instruments, Columbia, MD, USA) equipped with a diamond crystal. The spectrum represents the average of 32 scans acquired at a resolution of 2 cm^–1^ over the 4000–500 cm^–1^ wavenumber range at room temperature.

#### 2.3.2 Circular Dichroism

Circular dichroism (CD) spectroscopy was used to assess the secondary and tertiary structures of the isolated collagen. Measurements were carried out on a J1500 CD spectrometer (Jasco, Tokyo, Japan) using a quartz cylindrical cuvette with a 0.5 mm optical path length (Hellma Analytics, Hellma, Germany). For each analysis, the cuvette was loaded with 50 μL of collagen solution (10 mg/mL in deionized water). CD spectra were recorded by scanning continuously from 180 to 240 nm, at a rate of 50 nm/min, and at different temperatures (temperature ramp from 10 to 100 °C, recording at 5 °C intervals).

#### 2.3.3 Sodium Dodecyl Sulfate-Polyacrylamide Gel Electrophoresis (SDS-PAGE)

To assess the molecular weight distribution and purity of the isolated collagen, SDS–PAGE analysis was conducted. Freeze-dried collagen was dissolved in deionized water at concentrations of 1, 0.5, and 0.25 mg/mL, subsequently combined with loading buffer in a 1:1 ratio, and then heated at 90 °C for 5 min to promote protein denaturation. The electrophoresis setup consisted of a precast polyacrylamide gel (Bolt Bis-Tris Plus 8%, Invitrogen, Waltham, MA, USA). Each lane was loaded with 20, 10, and 5 μg of *C. reniformis* collagen alongside 4 μL of a prestained protein ladder (PageRuler Prestained Protein Ladder, 10–250 kDa; Thermo Fisher Scientific, Waltham, MA, USA) and 20 μg of bovine collagen type I. Following separation at 100 V, glycosylated proteins were visualized using the Pierce Glycoprotein Staining Kit (Thermo Fisher Scientific) according to the supplier’s protocol.

#### 2.3.4 Thermal Analysis

To determine the denaturation temperature of the isolated collagen, a microcalorimeter (Microcalvet, Setaram, Geneva, Switzerland) was employed. For the analysis, the sample cell was loaded with 500 μL of collagen solution (20 mg/mL in deionized water), while the reference cell was loaded with 500 μL of deionized water. The thermal program used consisted of a stabilization step (5 min at 5 °C), followed by a heating step from 5 to 90 °C (1 °C/min) and finally a cooling step from 90 to 5 °C (2 °C/min). The calorimetric output was tracked in real time with data points recorded every 0.4 s for subsequent analysis, and the results represent the average of three consecutive scans.

### 2.4 Marine Collagen-Based Scaffolds Production

The marine collagen-based scaffolds were produced by freeze-drying. Freeze-dried *C. reniformis* collagen was resuspended in ultrapure water at 50 mg/mL, and the three cross-linking agent solutions were prepared: 30 mM EDC, 50 μM GA, and 5 mM genipin. Collagen and cross-linking agent solutions at a ratio of 19:1 (v/v), respectively, were completely mixed using a magnetic stirrer (FB15001, Thermo Fisher Scientific) and poured into round silicon molds (⌀6 × 6 mm^3^), incubated at 4 °C overnight, and placed at −20 °C for 24 h. To remove any eventual residual cross-linking agents, the scaffolds were thoroughly washed with PBS and then freeze-dried (LyoAlfa 10/15, Azbil Telstar, Barcelona, Spain). The cross-linking approaches are illustrated in Figure 1

**Figure 1:**
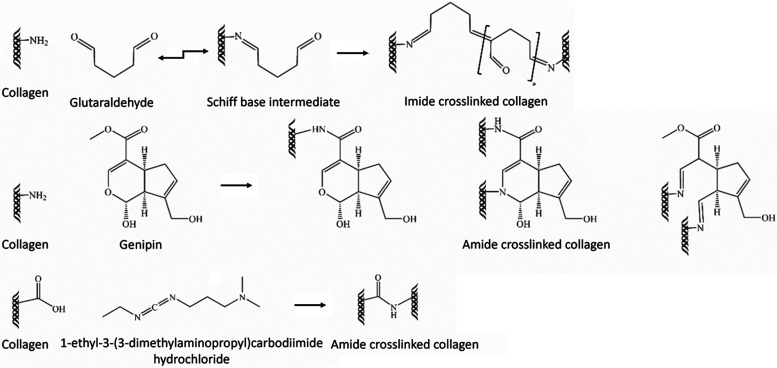
Schematic representation of collagen cross-linking reactions induced by glutaraldehyde (GA), genipin, and 1-ethyl-3-(3-(dimethylamino)­propyl)­carbodiimide hydrochloride (EDC) cross-linking agents. Adapted from ref 30

### 2.5 Marine Collagen-Based Scaffold Characterization

#### 2.5.1 Scanning Electron Microscopy (SEM)

To evaluate microstructure and surface morphology, the marine collagen-based scaffolds were observed by analytical scanning electron microscope (JSM-6010 LV, JEOL, Tokyo, Japan), using a beam energy of 10 keV at various levels of magnification. The scaffolds were sputter-coated with gold using the Leica EM ACE600 sputter coater (Leica, Wetzlar, Germany) before starting the SEM analysis. SEM analysis was performed with cross-sectioned scaffolds to observe the internal structure.

#### 2.5.2 Microcomputed Tomography (μCT)

Microcomputed tomography (μCT) was performed to analyze the scaffolds’ morphological features and to determine morphometric properties such as porosity (%), pore size (μm), and pore interconnectivity (%). A high-resolution μCT SkyScan 1217 (Bruker, Billerica, MA, USA) was employed for scanning scaffolds (*n* = 3). The X-ray source was set at 50 kV of energy and a current of 200 μA. Representative data was reconstructed using nRecon software (Bruker), while CT-Analyzer software (Bruker) was used to reslice all of the files of each sample and determine the quantitative morphometric parameters. The CTVox program (Bruker) was used for the 3D image reconstruction.

#### 2.5.3 Rheological Properties

To assess the mechanical properties of the scaffolds, a rheometer (Kinexus Prot, Malvern Instruments, Worcestershire, U.K.) was used for carrying out oscillatory studies. In the oscillatory mode, frequency sweep assays were performed, maintaining the temperature at 20 °C throughout the measurement period. The frequency was swept, from 0.1 to 10 Hz, to determine the elastic storage modulus (G′) and the viscous loss modulus (G″), with records taken every 6 points per frequency sweep range. All of the measurements were conducted within the range of the Linear Viscoelastic Region (LVER) previously determined. All plots were obtained by the average of at least 3 experiments.

#### 2.5.4 Swelling/Water Absorption Assays

To determine the scaffolds’ water absorption capacity, three samples (*n* = 3) of each formulation were used. First, all samples were weighted (*W_i_*) and then immersed in PBS at 37 °C. At 1, 24, 48, and 72 h, samples were gently blotted with filter paper to remove the superficial water and weighed (*W*_t_). The percentage of swelling was calculated using the following equation

1 $$\%\mathrm{swelling}=\left(\frac{W_{t}-W_{i}}{W_{i}}\right)\times 100$$

#### 2.5.5 Degradation Assay

To characterize the enzymatic degradation profile of scaffolds, three samples (*n* = 3) of each formulation were weighed (*W_i_*) and fully immersed in PBS containing 2 μg/mL collagenase A (Roche Diagnostics, Basel, Switzerland) at 37 °C for a specific period of time (1, 2, 3, 7, 14, and 21 days). At each time point, the scaffolds were rinsed with distilled water, freeze-dried, and weighed (*W*_t_). The degradation rate was estimated as the percentage of mass loss, determined using the following equation

2 $$\%\;\mathrm{mass}\;\mathrm{loss}=\left(\frac{W_{i}-W_{t}}{W_{i}}\right)\times 100$$

### 2.6 *In Vitro* Evaluation of Genipin-Cross-Linked Marine Collagen-Based Scaffolds

#### 2.6.1 Cell Culture (ATDC5, BJ, EA.hy926, ASCs)

Murine chondrogenic cells (ATDC5; ECACC 99072806) were expanded in Dulbecco’s Modified Eagle Medium (DMEM)/Ham’s Nutrient Mixture F12 supplemented with 1.1 g/L sodium bicarbonate, 10% fetal bovine serum (FBS) (Thermo Fisher Scientific), and 1% penicillin/streptomycin (Thermo Fisher Scientific). Human skin fibroblast cells (BJ; ATCC CRL-2522) were expanded in Eagle’s minimum essential medium (EMEM) supplemented with 2.2 g/L sodium bicarbonate, 0.11 g/L sodium pyruvate, 10% FBS (Thermo Fisher Scientific), and 1% penicillin/streptomycin (Thermo Fisher Scientific). Human umbilical vein cells (EA.hy926; ATCC CRL-2922) were expanded in DMEM supplemented with 1.5 g/L sodium bicarbonate, 0.11 g/L sodium pyruvate, 10% FBS (Thermo Fisher Scientific), and 1% penicillin/streptomycin (Thermo Fisher Scientific). Human adipose stem cells (ASCs) were isolated from the stromal vascular fraction of subcutaneous adipose tissue samples of patients submitted to liposuction procedures after informed consent for inclusion before participating in the study. The protocol was established with the Plastic Surgery Department of Hospital da Prelada (Porto, Portugal).^31,32^ The protocol was approved by the Hospital da Prelada Ethics Committee (PI N◦ 005/2019) and by the 3B’s Research Group. After isolation, cells were characterized by flow cytometry (FACSCalibur flow cytometer, BD Biosciences, USA) for stem cell surface markers to assess stemness.^33^ Briefly, cells were resuspended in a 3% v/v bovine serum albumin solution, incubated for 30 min at RT with antihuman rat antibodies, and then separated using the positive surface markers CD105, CD73, and CD90 and the negative markers CD45 and CD34 (BD Biosciences, Germany) (Supporting Information, Figure S1). Additionally, after each cell isolation procedure, the assessment of differentiation potential is routinely performed following established protocols.^34^ ASCs were expanded in Minimum Essential Medium α (α-MEM) (Thermo Fisher Scientific) supplemented with 2.2 g/L sodium bicarbonate, 10% FBS (Thermo Fisher Scientific), and 1% penicillin/streptomycin (Thermo Fisher Scientific) until passage 3. All cells were maintained in a 37 °C humidified incubator with 5% CO_2_, and the culture medium was changed every 2–3 days.

#### 2.6.2 Cytotoxicity Assessment

Before confluence, cells were trypsinized with TryplE Express (Thermo Fisher Scientific) and a seeding density of 3 × 10^5^ cells per scaffold was employed based on a top-down approach with a 50 μL droplet without additional medium, aiming to increase seeding efficacy. After 1 h of incubation, culture medium was added to complete a final volume of 500 μL. Prior to cell seeding, the scaffolds were immersed in 70% ethanol for 30 min to avoid microbial contamination and then rinsed twice with sterile PBS to remove all ethanol traces. Then, to assess the potential cytotoxicity of the developed scaffolds, the biological activity of ATDC5, BJ, EA.hy926, and ASCs cultured in the structures was evaluated 1, 7, and 14 days after cell seeding.

Cell viability was assessed by a resazurin-based assay (alamarBlue Cell Viability Reagent, Thermo Fisher Scientific), which quantitatively measures cellular metabolic activity. At the indicated time points, alamarBlue reagent was added (final dilution of 1:10 in the wells), and the cells were incubated for 3 h at 37 °C. Metabolically active cells reduced the nonfluorescent resazurin to the fluorescent resorufin, which was spectrofluorimetrically detected by 530 nm excitation and 590 nm emission using a microplate reader (Synergy HT, Biotek, Winooski, VT, USA). A blank subtraction was performed to account for any background reduction of resazurin occurring in medium-only wells (without cells) treated with alamarBlue.

Cell proliferation was evaluated by dsDNA quantification using the Quant-iT PicoGreen dsDNA assay (Thermo Fisher Scientific), in which the amount of dsDNA is directly proportional to cell number. During each culture at the determined time points, samples were washed with a PBS solution and digested with 0.05% collagenase (w/v) to ensure the complete removal of cells from the collagen matrix. Then, 1 mL of ultrapure water was added to each sample, which were maintained at 37 °C for 1 h and further kept at −80 °C. For analysis, the samples were thawed at RT and sonicated for 15 min in a water bath to complete the lysis of the cell membrane while avoiding DNA degradation. Fluorescence was measured using an excitation/emission wavelength of 485/528 nm, respectively, in a microplate reader (Synergy HT, Biotek, Winooski, VT, USA). The quantification of DNA was calculated based on a standard curve for DNA prepared by using DNA standards between 0 and 2 μg/mL.

Live/dead assay was performed to qualitatively assess cell viability at the indicated time points. Briefly, cells were incubated with Calcein AM (CA, 4 μg/mL, Thermo Fisher Scientific) and propidium iodide (PI, 1 μg/mL, Thermo Fisher Scientific). Calcein AM is hydrolyzed within live cells, generating green fluorescence, while PI binds to the nucleic acids of cells with compromised membranes (dead), emitting red fluorescence. After staining, samples were immediately examined using a Zeiss Axio Imager Z1 fluorescence microscope (Carl Zeiss, Jena, Germany).

Cell morphology was evaluated using rhodamine phalloidin (Pha, 228 μg/mL) and diamidino-2-phenylindole (DAPI, 10 μg/mL) staining. Cell membranes were permeabilized with Triton X-100 (0.2%) for 5 min, and then, the cytoplasm and nuclei were labeled with Pha and DAPI, respectively, for 20 min. Samples were immediately examined using a laser scanning confocal microscopy imaging system (TCS SP8, Leica, Wetzlar, Germany) using an excitation wavelength of 561 nm for Pha and 405 nm for DAPI.

### 2.7 Human Adipose Stem Cell Chondrogenic Differentiation

#### 2.7.1 Cytotoxicity Assays

To determine their potential in promoting chondrogenic differentiation, the genipin-cross-linked scaffolds were seeded with 2 × 10^5^ cells and cultured in basal or chondrogenic conditions. In basal conditions, cells were maintained with the same medium used for ASC expansion and in chondrogenic conditions, cells were maintained in α-MEM medium supplemented with insulin-transferrin-selenium-G supplement (Thermo Fisher Scientific), 1 mM dexamethasone, 0.1 M sodium pyruvate (Thermo Fisher Scientific), 0.17 mM ascorbic acid-2-phosphate (FUJIFILM Wako Pure Chemical Corporation, Richmond, VA, USA), and 0.35 mM l-proline and 10 ng/mL TGF-β3.^35^ Cell metabolic activity and proliferation of ASCs under basal or chondrogenic conditions were determined 1, 7, 14, and 21 days after cell seeding, as described above (2.5.2).

#### 2.7.2 RNA Isolation and Quantitative Real-Time Polymerase Chain Reaction (qPCR)

To evaluate the expression of chondrogenic differentiation-related genes of ASCs under basal or chondrogenic conditions, qPCR was carried out 1, 7, and 21 days after cell seeding. Briefly, after each culture period, all scaffolds were washed with PBS, digested with 0.05% collagenase (w/v) to ensure cell removal from the scaffolds, immersed in TRI reagent (Thermo Fisher Scientific), and stored at −80 °C until further use. RNA extraction was performed according to the manufacturer’s instructions, and then RNA concentration and purity were determined by UV spectroscopy (NanoDrop1000, Thermo Scientific). RNA was reverse-transcribed into cDNA according to the qScript cDNA Synthesis Kit (Quantabio, Beverly, MA, USA) protocol. The obtained cDNA was used as a template for the amplification of the target genes shown in Table 1, according to the manufacturer’s instructions of the PerfeCtaTM SYBR Green system (Quantabio) in a QuantStudio 5 Real-Time PCR System thermocycler (Applied Biosystems, Waltham, MA, USA). The following cycler conditions were used: 2 min at 95 °C and 45 cycles of 15 s at 95 °C, 30 s at Tm (°C) (melting temperature depending on the gene), and 30 s at 72 °C. B2-microglobulin (B2M) was used as a reference gene, and normalization was performed according to the Livak method (2^– ΔΔCT^ method), with day 1 of the basal condition (negative control) serving as calibrator.^35^

**Table 1:** Primer Sequences Used for qPCR Amplification^a^

Gene	Primer	Sequence (5′–3′)
*SOX9*	Forward	TTC ATG AAG ATG ACC GAC GC
	Reverse	GTC CAG TCG TAG CCC TTG AG
*ACAN*	Forward	TGA GTC CTC AAG CCT CCT GT
	Reverse	TGG TCT GCA GCA GTT GAT TC
*COMP*	Forward	TCT GCA TCA AAG TCG TCC TG
	Reverse	AGG GAT GGA GAC GGA CAT CAG
*2M*	Forward	TGG AGG CTA TCC AGC GTA CT
	Reverse	CGG ATG GAT GAA ACC CAG ACA

a SOX9–SRY-box transcription factor 9; *ACAN*–Aggrecan; *COMP*–Cartilage oligomeric matrix protein; *B2M*–β-2-microglobulin.

#### 2.7.3 Immunofluorescence Staining

The presence of chondrogenic-related proteins in ASCs maintained under basal and chondrogenic conditions was evaluated by immunohistochemistry, 21 days after cell seeding. Briefly, after each culture period, samples were washed with PBS, fixed with a 10% formalin solution, washed with PBS, and then incubated with 0.2% Triton X-100 in PBS for 10 min to increase cell membrane permeability. After washing, samples were maintained for 2 h in 3% (w/v) bovine serum albumin in PBS to block nonspecific antibody binding. Scaffolds were incubated with the primary antibodies anti-SOX9 and anti-ACAN (MilliporeSigma, Burlington, MA, USA) overnight at 4 °C and, after washing, with the respective secondary antibody: Alexa Fluor 488 (Santa Cruz Biotechnology, Dallas, TX, USA) and Alexa Fluor 594 (Thermo Fisher Scientific) for 2 h at RT. Additionally, the actin cytoskeleton was stained with Pha and nuclei with DAPI, 1 and 21 days after cell seeding. Secondary antibody-treated scaffolds were used as negative controls (Supporting Information, Figure S2). Samples were analyzed using a ZEISS LSM 980 (Carl Zeiss, Jena, Germany) fluorescence microscope.

### 2.8 Statistical Analysis

Data were presented as the mean ± standard deviation (SD) of at least three independent experiments. Statistical analyses were performed using GraphPad Prism 8.0.1 software (La Jolla, CA, USA). Data normality was evaluated by the Shapiro-Wilk test, and two-way ANOVA was performed, followed by Tukey’s test. Statistical significance was defined as a p-value less than 0.05 (*p* ≤ 0.05).

## 3 Results and Discussion

### 3.1 *C. reniformis* Collagen Characterization

Collagen is extensively employed in biomedical applications, making it essential to obtain a material of high purity and consistent quality.^6^ Before it can be applied in specific uses, the isolated collagen must demonstrate stable characteristics that meet the requirements of the intended application. Accordingly, the collagen isolated from *C. reniformis* was evaluated regarding its physicochemical properties.

The separation of the collagen’s chains according to their molecular weight was attempted by performing SDS-PAGE (Figure 2A). However, the samples remained in the high molecular weight region of the stacking gel, as they did not penetrate the separating gel, which has been observed in previous studies.^36,37^ This unusual behavior reflects the retention of collagen’s fibrillar structure throughout the isolation process, resulting in an apparent molecular weight of approximately 300 kDa.^38^ Furthermore, the glycoprotein stain dyed the *C. reniformis* collagen but not the mammalian collagen, further demonstrating the high glycosylation nature of the former, which is known to exhibit beneficial bioactivities such as antimicrobial and antioxidant.^39^ The FTIR spectrum revealed the presence of peaks related to amide A, B, I, II, and III bonds (Figure 2B), which are typically linked to collagen and provide insight into the secondary structure of various materials.^36,40^ The amide I band is the most intense and sensitive, making it a valuable indicator for examining the secondary structure of proteins.^41^ In contrast, the amide III band serves as a distinctive collagen signature, as it arises from the characteristic repeating tripeptide sequence Gly-X-Y.^41^ Additionally, the CD spectrum of the collagen presented a negative peak around 200 nm and a positive peak around 225 nm (Figure 2C). This is the distinctive pattern associated with the triple-helix structure of collagen,^42,43^ thus confirming that the protein was not denatured in the isolation process. The conservation of this conformation is paramount as its loss negatively impacts the functional performance of biological molecules.^44,45^ Overall, these results are in accordance with previously published *C. reniformis* collagen characterizations.^28,46^

**Figure 2:**
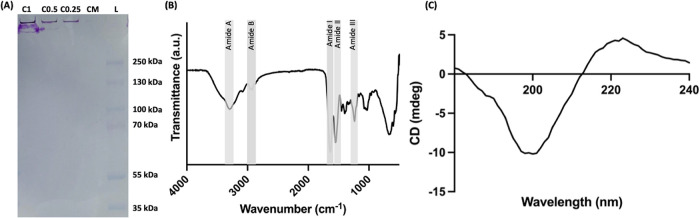
*C. reniformis* collagen characterization: (A) Sodium dodecyl sulfate-polyacrylamide gel electrophoresis (SDS-PAGE): C1: *C. reniformis* collagen (1 mg/mL); C0.5: *C. reniformis* collagen (0.5 mg/mL); C0.25 *C. reniformis* collagen (0.25 mg/mL); CM: bovine collagen type I; L: protein marker. (B) Fourier-transform infrared (FTIR) spectrum. (C) Circular dichroism (CD) spectrum.

Furthermore, a thermal analysis was conducted to determine the collagen’s denaturation temperature, which was found to be 60.8 ± 0.5 °C. Previous studies determined *C. reniformis* collagen thermal stability by differential scanning calorimetry and reported melting peaks between 69.2 and 82.1 °C.^47^ As collagen’s thermal stability is influenced by several factors, including its water content, extent of cross-linking, degree of glycosylation, and the pH of the surrounding environment,^48^ some batch-to-batch variation can be expected. To complement this result, the collagen CD spectrum was assessed at different temperatures, allowing us to determine that the average transition temperature that marks the onset of *C. reniformis* collagen denaturation occurs at 52.1 °C (Supporting Information, Figure S3). Nevertheless, considering that the denaturation temperature of marine-origin collagens is usually lower than vertebrate collagen, generally around 25–30 °C,^49,50^ *C. reniformis* collagen shows promise for the development of TE applications. This can be attributed to the high proline hydroxylation degree of *C. reniformis* collagen, around 44%,^28^ as well as to its isolation in the fibrillar form and high glycosylation degree.^47^ In fact, the denaturation process appears to occur in two distinct stages (Supporting Information, Figure S4), likely corresponding to the disruption of the fibrillar structure followed by the unfolding of the triple helix. Therefore, the denaturation of this collagen appears to be more complex than that typically observed in vertebrate collagen, warranting further investigation beyond the scope of the present study.

### 3.2 Marine Collagen-Based Scaffold Characterization

The detailed characterization of scaffolds aiming at TE strategies is essential, as their properties would influence the fate of seeded cells and thus determine their suitability for the intended application. To address the scaffolds’ morphological properties and the effect of the cross-linking reactions on the structural properties, microcomputed tomography (μCT) analysis was performed (Figure 3B). Although the different formulations presented a similar porosity and interconnectivity as well as a homogeneous distribution of the pores, their pore size and thickness were distinct. The EDC and GA formulations presented comparable pore size and thickness, both of which were lower than in the genipin formulation. Pore size can impact both the mechanical properties of the scaffolds and cell behavior, such as cell attachment, migration, and infiltration, and the optimal pore size for a specific application varies depending on the cell type. Generally, scaffolds with smaller pores (ø < 100 μm) present a high surface area for cell attachment but are challenging for cell penetration, while larger pores (ø > 100 μm) facilitate a higher cell seeding efficiency and migration to the inner regions of the construct.^51^ However, if the pores are too large, cells cannot bridge them and fail to completely populate the scaffold.^51^ Nevertheless, according to the literature, the pore size of the developed scaffolds could be suitable for the culture of several different cell types, such as fibroblasts, chondrocytes, and mesenchymal stem cells.^52–55^ Furthermore, the high porosity and interconnectivity of the developed structures, above 86 and 96%, respectively, showed promise for cellular studies as these features are essential for the transport of nutrients, removal of metabolic wastes, and facilitate cell proliferation and migration.^56,57^ Yang et al. employed similar cross-linking methods to develop gelatin scaffolds that presented lower porosities, ranging from 66.6 to 51.2%.^58^ In that regard, the currently developed structures demonstrate superior potential for TE applications. To further evaluate the scaffolds’ microstructure, SEM micrographs of the internal structure were acquired (Figure 3C). It is possible to observe that all formulations possessed a highly porous honeycomb-like ordered internal structure and that the pores of the genipin formulation were the largest, while the pore size of the EDC and GA formulations were comparable, in accordance with μCT results.

**Figure 3:**
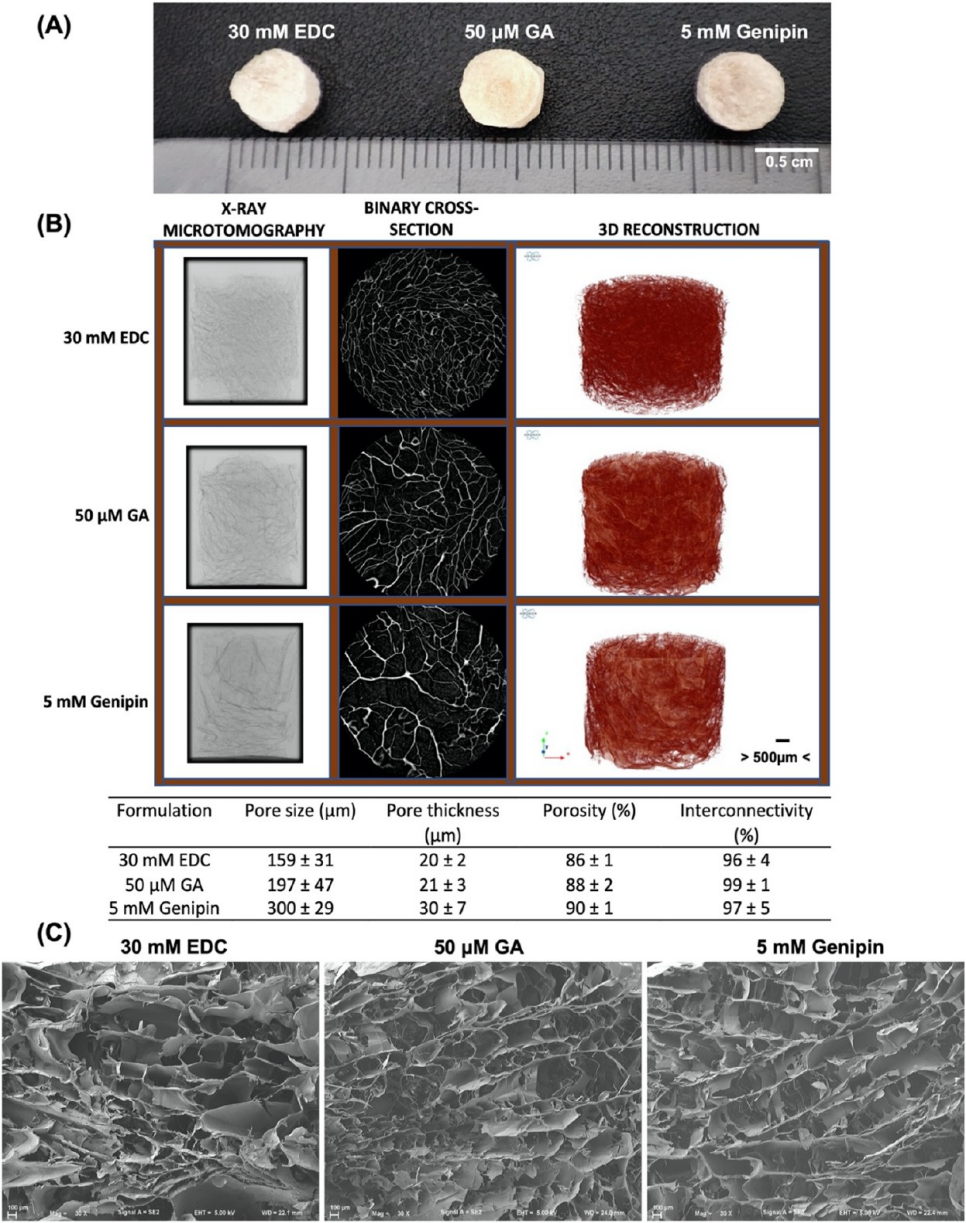
Assessment of the morphological and structural properties of the developed freeze-dried scaffolds. (A) Representative macroscopic images of scaffolds. (B) Representative images and microarchitectural features of scaffolds obtained by microcomputed tomography (μCT) analysis. Data are mean ± SD (*n* = 3). (C) Representative scanning electron microscope (SEM) micrographs of transversal sections of scaffolds (magnification 30×).

The viscoelastic properties of the developed freeze-dried scaffolds were assessed as a function of frequency through oscillatory shear rheometry assays (Figure 4A). The results were similar among all formulations, which exhibited an elastic modulus (G′) almost independent of frequency, with the curves practically parallel to the *x*-axis, almost up to 10 Hz, and higher than the viscous modulus (G″). This means that the scaffolds’ elasticity is more influential than their viscosity. According to the results, the storage modulus showed less susceptibility to frequency variations compared to the loss modulus. These findings indicate the high stability of the structures, suggesting that the cross-linking reactions were efficient, as the scaffolds maintained their cohesion when submitted to increasing frequencies. This behavior had been previously reported for both collagen-based and collagen/chitosan composite scaffolds.^59,60^ Furthermore, higher pore size did not impair the rheological performance of the genipin-cross-linked structures.

**Figure 4:**
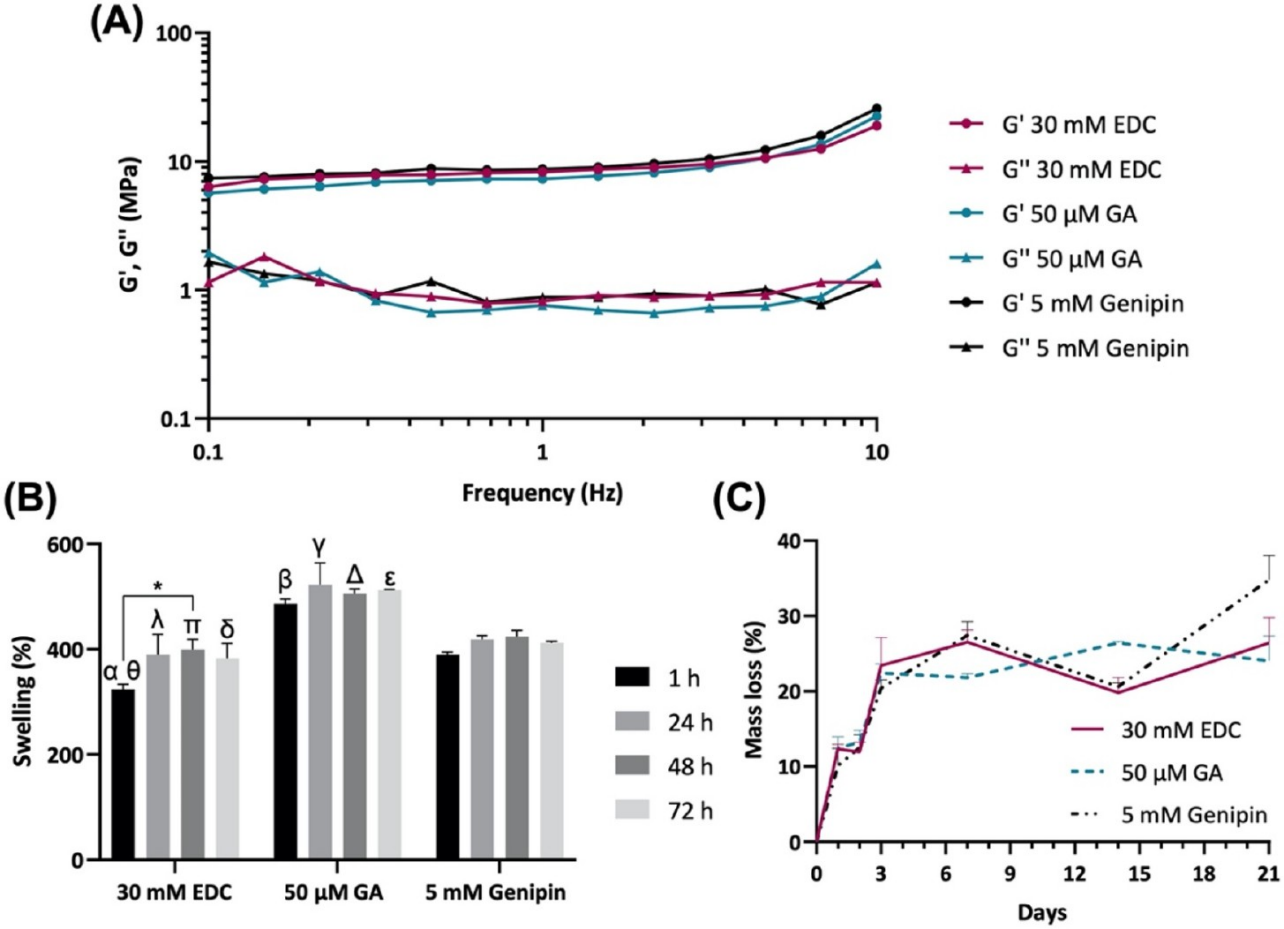
(A) Rheological properties of the *C. reniformis* collagen scaffolds determined by the oscillatory frequency test. (B) Swelling percentage of the *C. reniformis* collagen scaffolds immersed in PBS for 1, 24, 48, and 72 h. Data are mean ± SD (*n* = 3, statistical significance for * *p* ≤ 0.05, ** *p* ≤ 0.01, *** *p* ≤ 0.001, and **** *p* ≤ 0.0001, and symbols denote statistical differences: α (*) and β (**) compared with 1 h of 5 mM Genipin, γ (***) compared with 24 h of 5 mM Genipin, Δ (**) compared with 48 h of 5 mM Genipin, ε (**) compared with 72 h of 5 mM Genipin, Θ (****) compared with 1 h of 50 μM GA, λ (***) compared with 24 h of 50 μM GA, π (***) compared with 48 h of 50 μM GA, δ compared with 72 h of 50 μM GA). (C) Degradation of the *C. reniformis* collagen scaffolds immersed in PBS containing 2 μg/mL collagenase A for 1, 2, 3, 7, 14, and 21 days. Data are mean ± SD (*n* = 3).

The water uptake of the developed scaffolds was evaluated, and it was observed that all formulations presented high absorption capacity, ranging from 400 to 500% of swelling (Figure 4B). The genipin and GA formulations achieved equilibrium in the first hour of assay, while the EDC formulation took 24 h to achieve that state. Furthermore, in equilibrium, the swelling % of genipin and EDC formulations was similar, while the GA formulation presented the highest swelling. The values obtained were considerably higher than previously reported in other studies employing both gelatin and collagen/chitosan composite scaffolds cross-linked with genipin and GA.^59,61^ Moreover, the fast and considerable water uptake of the structures was enabled by their high pore size and interconnectivity and by the collagen hydrophilicity.^62,63^ Indeed, the scaffolds’ ability to absorb water is a critical property when evaluating their suitability for TE applications since higher swelling ratios are associated with increased cell adhesion and infiltration, having also a severe impact on other properties such as the mechanical features and molecular diffusion.^64^ Furthermore, from a clinical point of view, this property is crucial as, upon implantation, the construct should quickly absorb the fluids around the tissue and fill the defect properly.^65^ In this regard, although GA presented the highest water uptake, all formulations were suitable for maintaining the appropriate tissue hydration required for *in vitro* and *in vivo* assays.

The stability of the developed scaffolds was assessed by collagenase degradation to closely mimic the *in vivo* degradation conditions, thus providing insight into their expected behavior under physiological conditions (Figure 4C). It has been determined that the degradation profile of all formulations was similar, especially up to 3 days. At day 7, the GA formulation presented a slightly lower mass loss (20%) than the other formulations (25%), while at day 14, the profile inverted. Additionally, at day 21, the genipin formulation displayed the highest mass loss, around 35%, while the other formulations were close to 25%. Collagenase is known to induce collagen degradation by cleaving the peptide bonds between leucine and glycine triple-helical collagen.^66^ However, the intra- and intermolecular cross-linked collagen network hinders the collagenase access to the cleavage sites, thus increasing the biostability of cross-linked scaffolds. In this sense, the cross-linking reactions seemed to have been similarly effective. Furthermore, *C. reniformis* collagen fibrils tend to be intrinsically resistant to bacterial collagenase,^40^ which could enhance the effect of the cross-linkers. Gelatin scaffolds developed by Yang et al. employing the same cross-linkers degraded much faster, as it was reported that the GA formulation was the most stable and yet presented 60% of mass loss after only 6 h.^58^ In this sense, it is important to consider the stability of the construct to be implanted as it is crucial for a successful regeneration process, since if the scaffold is degraded too rapidly, cell adhesion and new tissue formation will be prevented. Taking this into account, the collagenase degradation test indicated that the cross-linking reactions were equally effective and that the developed collagen scaffolds seemed suitable for TE applications.

To perform the biological performance assessment, the most promising formulation for the development of TE applications was selected. The scaffold characterization determined that all formulations were mostly similar except for the genipin-cross-linked scaffolds, presenting the highest pore size, which is beneficial for cell migration and infiltration. Moreover, it has been previously demonstrated that the natural cross-linking agent genipin exhibits lower cytotoxicity when compared with conventional cross-linkers, namely GA and EDC.^67,68^ Therefore, the greener and environmentally friendly approach consisting of producing marine collagen-based scaffolds using a natural cross-linker, which also resulted in larger pores without other significant variations, was preferred.

### 3.3 Genipin-Cross-Linked Marine Collagen-Based Scaffolds *In Vitro* Evaluation

To guarantee that the processing method did not result in any harmful residual compounds, the cytotoxicity of genipin-cross-linked scaffolds was determined by assessing the *in vitro* biological performance of ASCs, ATDC5, BJ, and EA.hy926 cells cultured up to 14 days. The metabolic activity was determined by alamarBlue assay, and the results demonstrated that the developed scaffolds had no cytotoxic effect, as the metabolic activity was not detrimentally affected (Figure 5A). ASCs presented the highest cell metabolism at early time points, while by day 14, all cell types showed similar metabolic activity, with the exception of EA.hy926 cells, which displayed a slightly lower metabolic activity. Nonetheless, the cell metabolism of all cells studied significantly increased in a time-dependent manner. Additionally, DNA quantification was performed to evaluate cell proliferation (Figure 5B). An increase in cell number in all time points and cell types tested was observed, except for ASCs from day 1 to 7, possibly due to cells still adapting to the scaffold. BJ and EA.hy926 cells showed the lowest increase in cell number over time, although constantly proliferating as well, while at day 14, ASCs presented the highest DNA quantification results. Therefore, a significant increase in cell proliferation was observed in all cell types over time, similarly to the findings from cell metabolism results. Moreover, when compared to the results obtained in our lab using other scaffolds produced using similar procedures (i.e., freeze-drying of biopolymers solutions to render 3D porous structures), the herein reported DNA quantification results were in the same range, taking into consideration the size of the scaffolds and the seeding cell type and density, which illustrates well the biomedical potential of *C. reniformis* collagen scaffolds. In particular, ASCs cultured on scaffolds composed of shark collagen and hyaluronic acid showed a gradually decreasing DNA content, varying between 1000 and 500 ng/mL,^69^ whereas the herein accounted DNA content increased from ∼500 to ∼1800 ng/mL. Additionally, the significant increase observed herein, also with ATDC5 cells (∼300 to ∼1700 ng/mL), contrasts with previous results obtained using squid chitosan scaffolds, in which the number of cells declines in time (DNA content varying between ∼950 and ∼250 ng/mL).^70^

**Figure 5:**
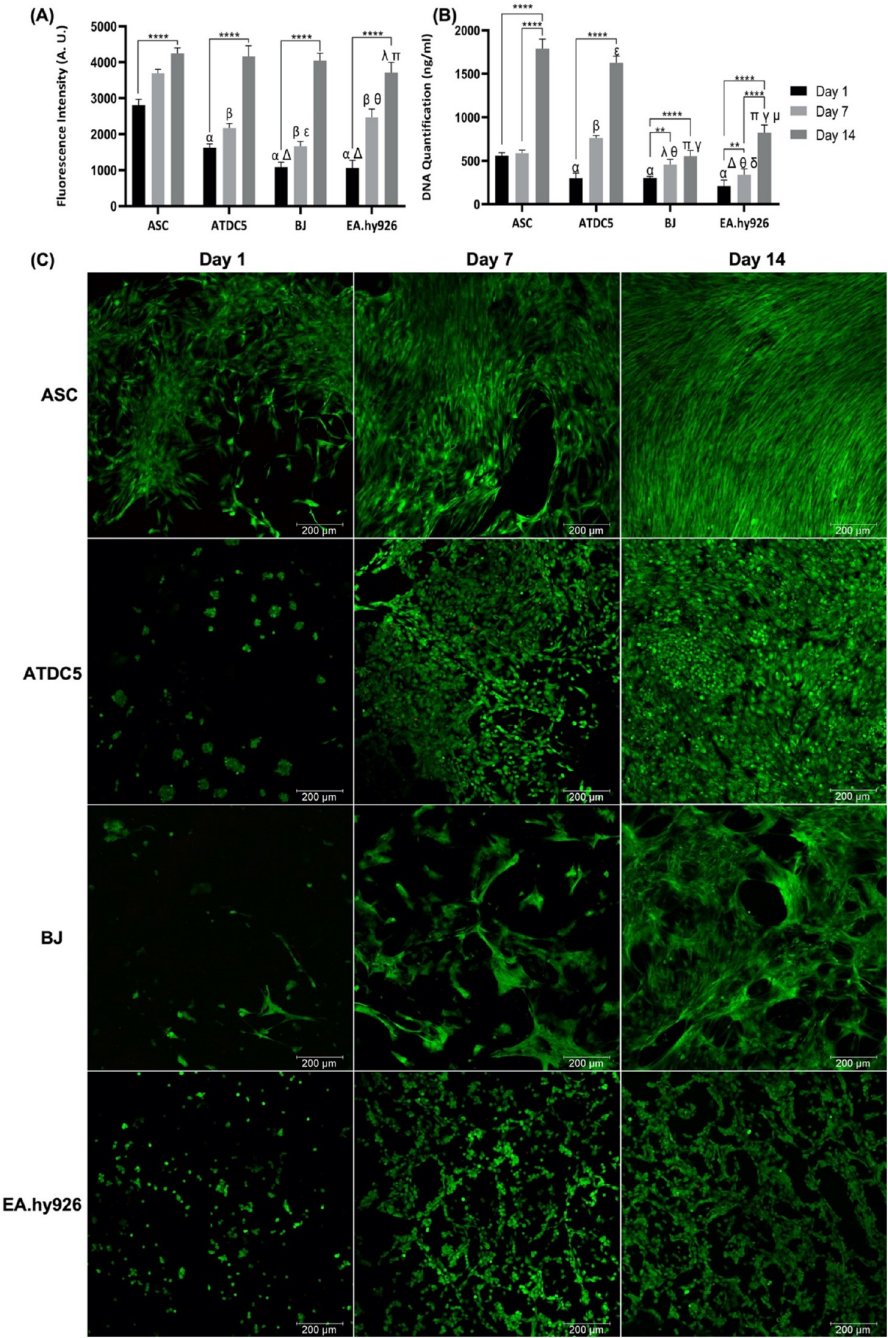
(A) Metabolic activity of ASCs, ATDC5, BJ, and EA.hy926 cells cultured in *C. reniformis* collagen scaffolds for 1, 7, and 14 days as determined by alamarBlue assay. Data are mean ± SD (*n* = 3, statistical significance for *** *p* ≤ 0.001 and **** *p* ≤ 0.0001, and symbols denote statistical differences: α (****) compared with day 1 of ASCs, β (****) compared with day 7 of ASCs, Δ (****) compared with day 1 of ATDC5, ε (****) compared with day 7 of ATDC5, θ (****) compared with day 7 of BJ, λ (****) compared with day 14 of ASCs, and π (***) compared with day 14 of ATDC5). (B) Cell proliferation of ASCs, ATDC5, BJ, and EA.hy926 cells cultured in *C. reniformis* collagen scaffolds for 1, 7, and 14 days as determined by dsDNA quantification. Data are mean ± SD (*n* = 3, statistical significance for * *p* ≤ 0.05, ** *p* ≤ 0.01, *** *p* ≤ 0.001 and **** *p* ≤ 0.0001, and symbols denote statistical differences: α (****) compared with day 1 of ASC, β (***), λ (**) and Δ (****) compared with day 7 of ASCs, ε (***) and π (****) compared with day 14 of ASCs, θ (****) compared with day 7 of ATDC5, γ (****) compared with day 14 of ATDC5, δ (*) compared with day 7 of BJ, and μ (****) compared with day 14 of BJ). (C) Microscopy of live/dead assay of ASCs, ATDC5, BJ, and EA.hy926 cells cultured in *C. reniformis* collagen scaffolds for 1, 7, and 14 days. Viable cells were stained with Calcein AM (green) and dead cells with PI (red). Scale bar: 200 μm.

To further validate the results, a live/dead assay was performed to determine cell viability (Figure 5C). It is possible to observe that the green signal, related to living cells, greatly increased over time in all cell types, demonstrating that the cells were alive and proliferating, in accordance with the alamarBlue and DNA quantification results. Moreover, at day 14, the green signal was more intense in ASCs and ATDC5 cells, which also displayed the highest DNA quantification results.

To complement the *in vitro* biological performance assessment, Pha/DAPI staining was performed to analyze cell morphology (Figure 6). Besides the cellular proliferation observed over time in all cell types, in line with the previously obtained results, cell spreading is also a common feature, thus demonstrating good adhesion to the substrate. BJ cells and undifferentiated ASCs exhibited their characteristic fibroblast-like morphology, while ATDC5 cells displayed a typical polygonal shape. Accordingly, previous studies have determined that dense collagen fiber networks supported a flattened cell shape of chondrocytes, in contrast with scaffolds composed of hyaluronic acid or synthetic polymers in which cells showed a mixed morphology of both round and elongated cells.^71–73^ In particular, the EA.hy926 cells seemed to assemble into linear cord-like vessels, a crucial step in the process of establishing a functional vascular network resembling an *in vitro* process of angiogenesis.^74^

**Figure 6:**
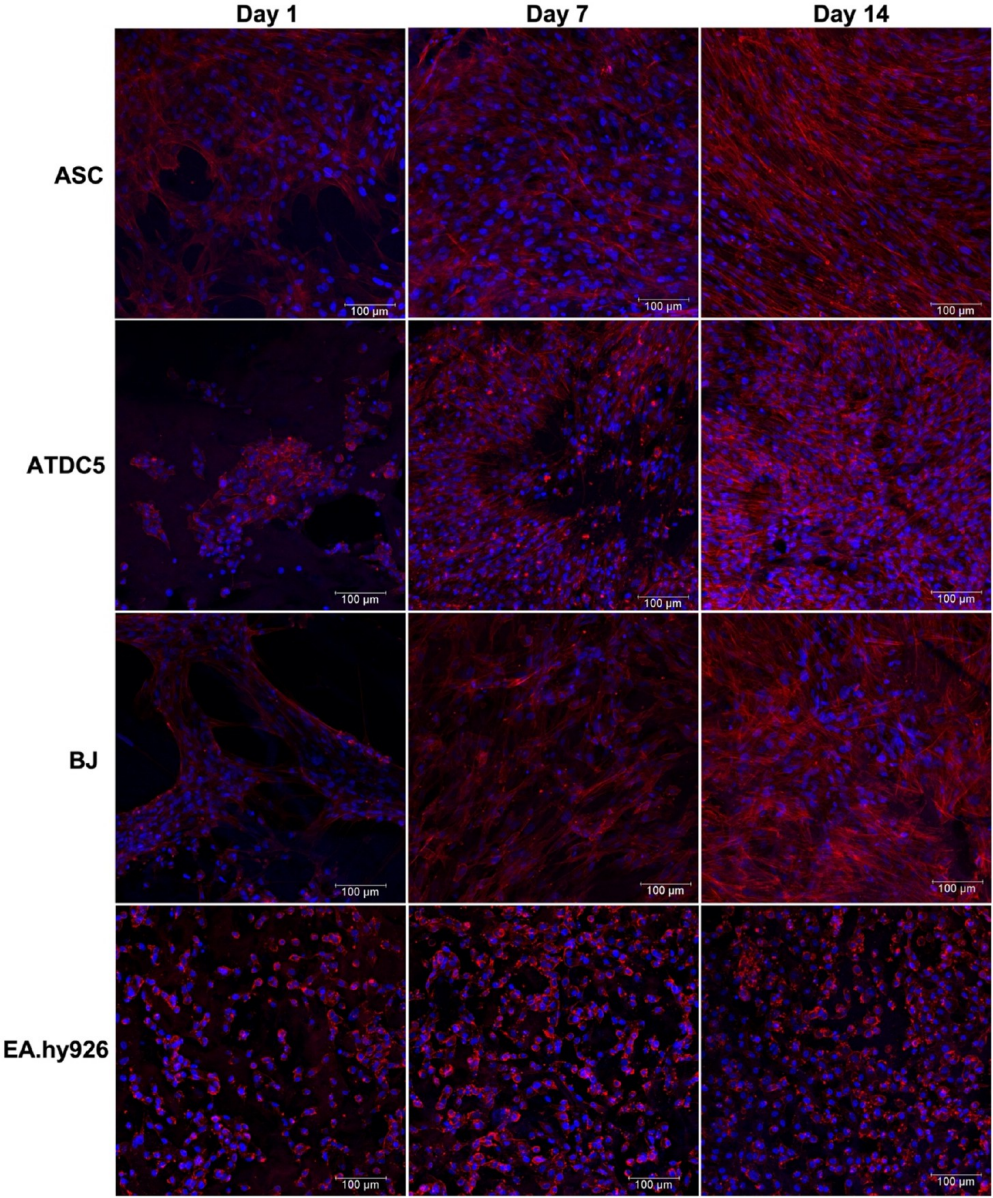
Fluorescence microscopy images of ASCs, ATDC5, BJ, and EA.hy926 cells cultured in *C. reniformis* collagen scaffolds for 1, 7, and 14 days, upon staining with Phalloidin (cytoskeleton, in red) and DAPI (nuclei, in blue). Scale bar: 100 μm.

Overall, these results confirm the positive effect of *C. reniformis* collagen on cellular activity, also demonstrated in previous works.^20,21,28^ However, despite having positive results with all cell types tested, the results with ASCs and ATDC5 cells were slightly better, suggesting that these marine collagen-based scaffolds exhibited appropriate features to be further considered for cartilage regeneration therapies, namely their combination with stem cells promoting differentiation toward chondrogenic lineage.

### 3.4 Human Adipose Stem Cell Chondrogenic Differentiation

After determining the cytocompatibility of the scaffolds, their potential to support stem cell chondrogenic differentiation was evaluated. Scaffolds maintained in basal medium without chondrogenic supplements, thus mimicking the natural articular cartilage microenvironment, were compared with scaffolds maintained in chondrogenic differentiation medium containing, among others, the growth factor TFG-β3 that is commonly employed as an inductor of stem cell chondrogenic differentiation.^75^ Both the metabolic activity and DNA quantification from ASCs cultured for 1, 7, 14, and 21 days were determined (Figure 7A,B). Under basal conditions, the results were similar to the previously obtained (and shown in Figure 5A,B), with an increase in cell metabolism and proliferation observed over time. However, under chondrogenic conditions, there was a constant decrease in cell metabolic activity and DNA content. The Pha/DAPI staining was performed to further validate these results, and cell proliferation was only observed over time under basal conditions, in accordance with DNA quantification data (Figure 7C). The absence of cell proliferation observed in chondrogenic medium could be attributed to the chondrogenic differentiation of ASCs, as it has been reported in other studies that DNA content decreases in the first weeks of *in vitro* chondrogenic differentiation.^69,76^ This is due to the transition from the proliferative stage to the differentiation and maturation stages of chondrocyte development that occurs during the early stages of chondrogenic differentiation.^77^ During this process, cells undergo cell cycle arrest, which is crucial for proper cartilage formation.^78^ Furthermore, during *in vitro* chondrogenic differentiation, cells may undergo apoptosis as part of the maturation process, thus leading to a reduction in the total DNA content of the culture.^77^

**Figure 7:**
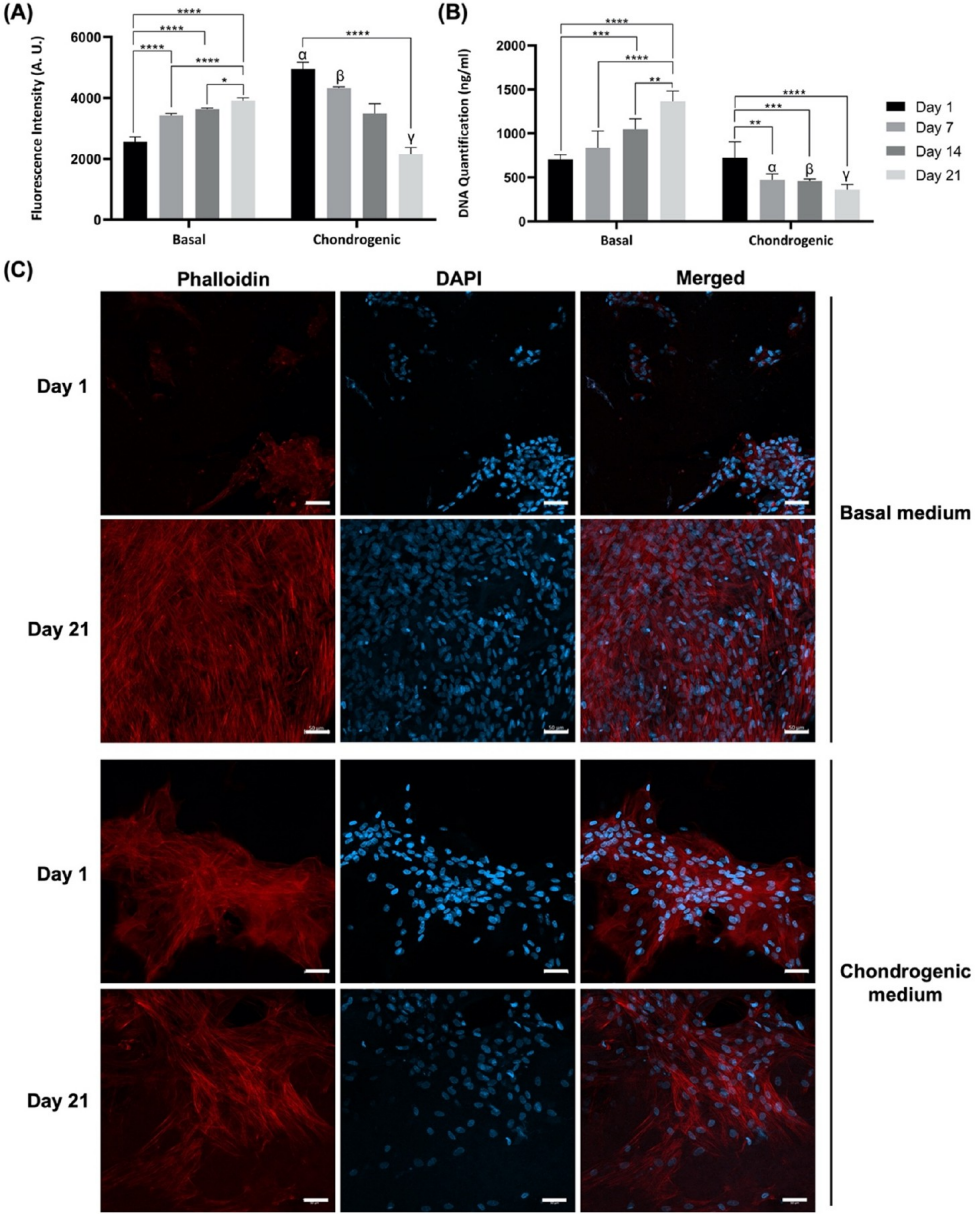
(A) Metabolic activity of ASCs cultured in *C. reniformis* collagen scaffolds under basal and chondrogenic conditions for 1, 7, 14, and 21 days as determined by alamarBlue. Data are mean ± SD (*n* = 3, statistical significance for * *p* ≤ 0.05, ** *p* ≤ 0.01, *** *p* ≤ 0.001, and **** *p* ≤ 0.0001, and symbols denote statistical differences: α (****) compared with day 1 of basal condition, β (****) compared with day 7 of basal condition, γ (****) compared with day 21 of basal condition). (B) Cell proliferation of ASCs cultured in *C. reniformis* collagen scaffolds under basal and chondrogenic conditions for 1, 7, 14, and 21 days as determined by dsDNA quantification. Data are mean ± SD (*n* = 3, statistical significance for ** *p* ≤ 0.01, *** *p* ≤ 0.001, and **** *p* ≤ 0.0001, and symbols denote statistical differences: α (****) compared with day 7 of basal condition, β (****) compared with day 14 of basal condition, and γ (****) compared with day 21 of basal condition). (C) Fluorescence microscopy images of ASCs cultured in *C. reniformis* collagen scaffolds under basal and chondrogenic conditions for 1 and 21 days upon staining with Phalloidin (cytoskeleton, in red) and DAPI (nuclei, in blue). Scale bar: 50 μm.

To evaluate chondrogenic differentiation, gene expression of relevant chondrogenesis-related markers *SOX9*, aggrecan (*ACAN*), and cartilage oligomeric matrix protein (*COMP*) was evaluated after 1, 7, and 21 days of cell culture by RT-PCR (Figure 8A). *SOX9* is a member of the Sox (Sry-type HMG box) gene family predominantly expressed in cartilage, essential for chondrocyte differentiation and cartilage formation by inducing the expression of cartilage-related genes such as *ACAN*.^75,79^ ACAN is a cartilage ECM proteoglycan and COMP is a cartilage protein involved in regulating the macromolecular assemblies within the cartilage ECM components, and their upregulation is indicative of chondrogenic differentiation.^80^ The expression of these three markers followed a similar trend under both basal and chondrogenic conditions, as generally there was a slight decrease from day 1 to 7, followed by an increase at day 21. Additionally, it is noteworthy that the expression of *ACAN* and *COMP* was noticeably upregulated at day 21 under chondrogenic conditions compared with basal conditions, roughly 2-fold and 30-fold, respectively. Such a tendency was also observed in *SOX9* expression, although not so pronounced. The high expression levels of these chondrogenesis-related markers at day 21 strongly suggested the possibility of ongoing functional development of cells and cartilage-like ECM synthesis. Additionally, it has been described that the expression of *SOX9*, *ACAN*, and *COMP* during chondrogenic differentiation of ASCs typically occurs at specific time points. Previous studies indicated that the onset of chondrogenic differentiation is marked by an increase in *SOX9* gene expression, which reaches its maximum level at day 21.^75^ Similarly, *ACAN* and *COMP* gene expression is upregulated at day 21, with the expression of these genes increasing from day 7 to day 21, thus being significantly higher at posterior time points.^81^ Therefore, the results obtained in the present work are in line with the literature stating that the expression of *SOX9*, *ACAN*, and *COMP* is particularly observed during the later stages of chondrogenic differentiation.

**Figure 8:**
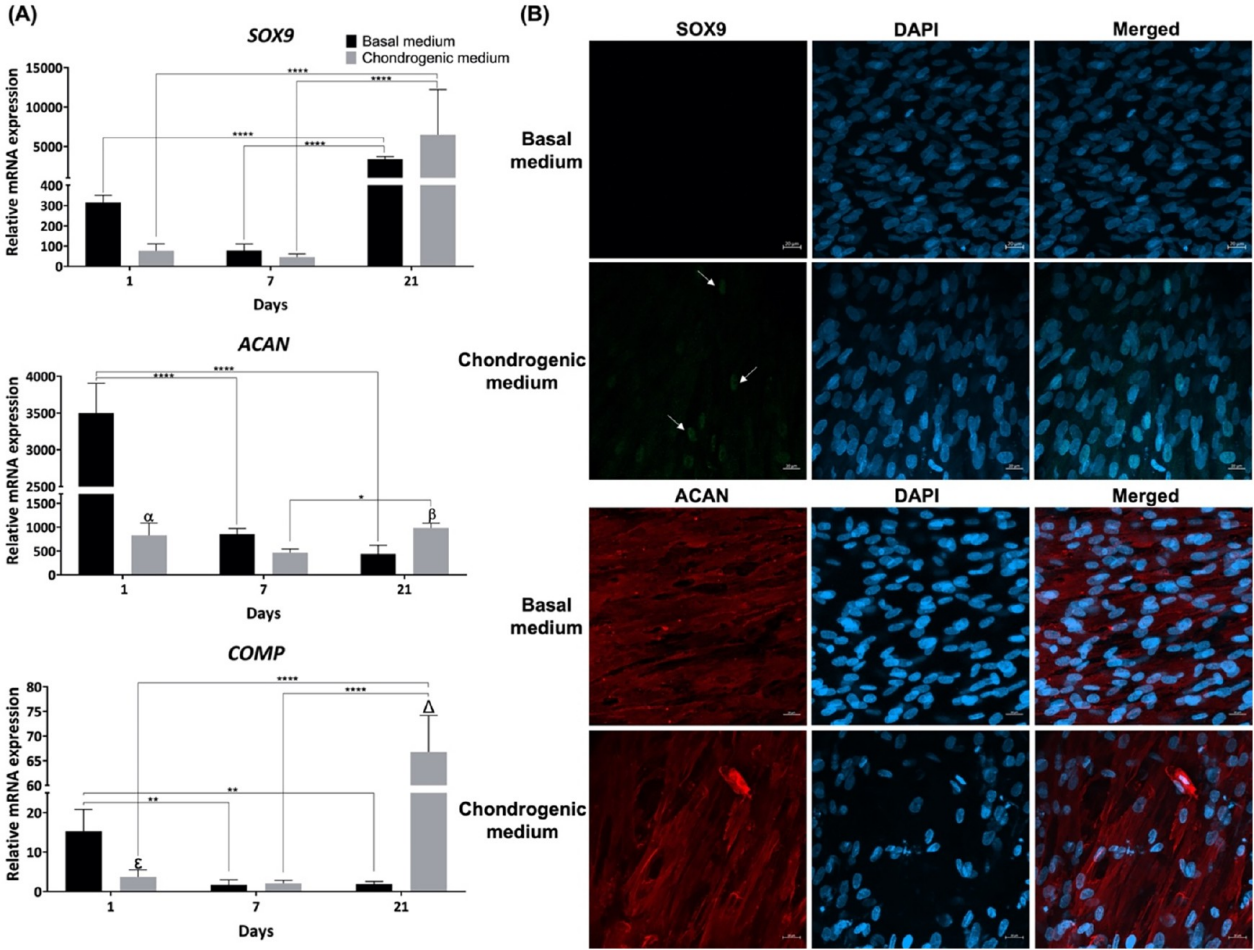
(A) Quantitative gene expression encoding chondrogenesis-related markers (SOX9, ACAN, and COMP) by ASCs cultured in *C. reniformis* collagen scaffolds under basal and chondrogenic conditions for 1, 7, and 21 days as determined by qPCR. Data are mean ± SD (*n* = 3, statistical significance for * *p* ≤ 0.05, ** *p* ≤ 0.01, and **** *p* ≤ 0.0001, and symbols denote statistical differences: α (****) compared with day 1 of ACAN basal condition, β (*) compared with day 21 of ACAN basal condition, ε (*) compared with day 1 of COMP basal condition, and Δ (****) compared with day 21 of COMP basal condition). (B) Immunofluorescence detection of representative chondrogenesis-related markers SOX9 (green, white arrows) and ACAN (red) by ASCs cultured in *C. reniformis* collagen scaffolds under basal and chondrogenic conditions for 21 days upon staining with DAPI (nuclei, in blue). Scale bar: 20 μm.

To further complement these results, the immunodetection of SOX9 and ACAN was assessed after 21 days of culture (Figure 8B). The immunofluorescence images showed protein expression of both chondrogenesis-related markers under chondrogenic culture conditions, while under basal conditions, only the expression of ACAN was observed. These observations were mostly consistent with the results of gene expression of chondrogenesis-related genes. The only exception was the lack of SOX9 protein expression under basal conditions, since gene expression was detected. However, the relationship between gene expression and protein expression is complex and not always directly correlated since it can be influenced by several processes, such as post-transcriptional, translational, and degradation regulation mechanisms.^82^ These mechanisms, which range from mRNA or protein degradation to mRNA translation inhibition, are known to lead to stronger deviations from the ideal mRNA–protein correlation, particularly during dynamic phases, such as cellular differentiation.^82,83^ Moreover, there is a delay between transcriptional induction and increased protein level as maturation, export, and translation of mRNA takes time.^83^ Taking this into account, it can be hypothesized that under basal culture conditions, ASCs’ chondrogenic differentiation was also taking place, although slower than under chondrogenic conditions. Therefore, it can be determined that in an early culture period, *C. reniformis* collagen-based scaffolds seem to support chondrogenic differentiation of ASCs. In fact, these results were comparable to *in vitro* studies performed using other collagen-based matrices, namely, mammalian collagen-based and shark collagen/hyaluronic acid composite scaffolds.^69,71^ In the latter, chondrogenic differentiation of ASCs was also achieved without the addition of any exogenous induction component.^69^ Furthermore, comparing our data with other works, it can be established that collagen-based scaffolds provide higher cell adhesion and chondrogenic differentiation of stem cells than synthetic polymers or genipin-cross-linked gelatin scaffolds.^71,84^

## 4 Conclusions

Collagen was produced from *C. reniformis* in fibril form, glycosylated, exhibiting a high denaturation temperature, thus promising for biomedical applications. Stable freeze-dried *C. reniformis* collagen scaffolds were successfully developed using EDC, GA, and genipin as cross-linking agents. A thorough physicochemical characterization was performed, demonstrating that all formulations presented similar rheological properties and enzymatic degradation rates. However, there were dissimilarities regarding the swelling rate and microstructure, as the GA formulation presented the highest swelling capacity, while genipin-cross-linked scaffolds had the largest pore size, the latter being favorable for cell proliferation. Taking that into account, along with literature stating its low cytotoxicity, genipin genipin-containing formulation was selected to further determine its biological performance with ATDC5, BJ, EA.hy926 cell lines, mimicking cartilage, skin, and vascular endothelial cells, respectively, as well as with ASCs. *In vitro* studies determined that these structures successfully enhanced cell metabolic activity and proliferation in a time-dependent manner up to 14 days, validating their suitability for regeneration therapies. Furthermore, *in vitro* differentiation of ASCs into chondrogenic lineage was successfully achieved with and without exogenous induction, demonstrated by the gene and protein expression of markers such as SOX9, ACAN, and COMP, indicating the potential of these structures to be explored as alternatives to commercially available matrices for cartilage repair. Therefore, it was established that the sustainably obtained *C. reniformis* collagen presents great biological properties, thus being a versatile and suitable option for the future development of several TE approaches, with a particular focus on cartilage regeneration. Further studies, including *in vivo* testing using the developed scaffolds, should be performed to confirm their potential prior to finding a clinical applicability.

## Supplementary Material



## Data Availability

The data that support the findings of this study are available in the manuscript or upon reasonable request from the authors.

## Notes

Flow cytometry analysis of undifferentiated ASCs employed in this study, immunofluorescence micrographs of secondary antibody-treated scaffolds used as negative controls, variation of the maximum ellipticity of *C. reniformis* collagen CD spectrum at 225 nm in relation to temperature, and a representative graph of the thermal analysis of *C. reniformis* collagen (PDF)

## ∥

CBQF—Centro de Biotecnologia e Química Fina—Laboratório Associado, Escola Superior de Biotecnologia, Universidade Católica Portuguesa, Rua de Diogo Botelho 1327, 4169–005 Porto, Portugal
