# Supplementary material for: Unraveling the Potential of Chondrosia reniformis Collagen for Tissue Engineering Scaffolds, with Particular Insights into Chondrogenic Differentiation
Source: Biomacromolecules. 2026 Jan 8;27(2):1122–37. doi: 10.1021/acs.biomac.4c01793 (PMC12892333; doi:10.1021/acs.biomac.4c01793)
Supplement: Supplementary file 1 [file bm4c01793_si_001.pdf]

Unraveling the potential of *Chondrosia reniformis* collagen for tissue engineering scaffolds, with particular insights into chondrogenic differentiation

Miguel S. Rocha [1,2], Ana C. Carvalho [1,2], Catarina F. Marques [1,2], Filipa Carneiro [1,2], Rita O. Sousa [1,2], Eva Martins [1,2†], Eleonora Tassara [3], Rui L. Reis [1,2], Tiago H. Silva [1,2*]

[1] 3B's Research Group, I3Bs – Research Institute on Biomaterials, Biodegradables and Biomimetics, University of Minho, Headquarters of the European Institute of Excellence on Tissue Engineering and Regenerative Medicine, AvePark, Parque de Ciência e Tecnologia, Rua Ave 1, 4805-694 Barco, Guimarães, Portugal;

[2] ICVS/3B's–PT Government Associate Laboratory, Braga/Guimarães, Portugal;

[3] Department of Earth, Environment and Life Sciences (DISTAV), University of Genova, Via Pastore 3, 16132 Genova, Italy;

* Author to whom correspondence should be addressed.

[†] Current address: CBQF—Centro de Biotecnologia e Química Fina—Laboratório Associado, Escola Superior de Biotecnologia, Universidade Católica Portuguesa, Rua de Diogo Botelho 1327, 4169-005 Porto, Portugal.

Supplementary information

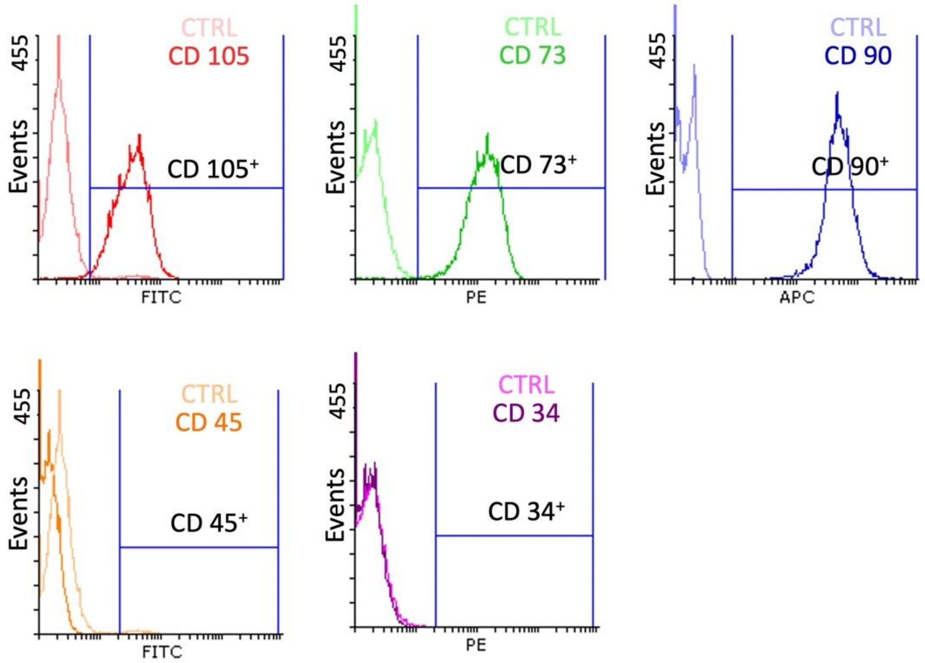

Figure S1 - Flow cytometry analysis displaying positive expression of mesenchymal stem cell (MSC) surface markers (CD105, CD73, and CD90) and negative expression of hematopoietic markers (CD45 and CD34) in undifferentiated human MSCs, derived from adipose tissue.

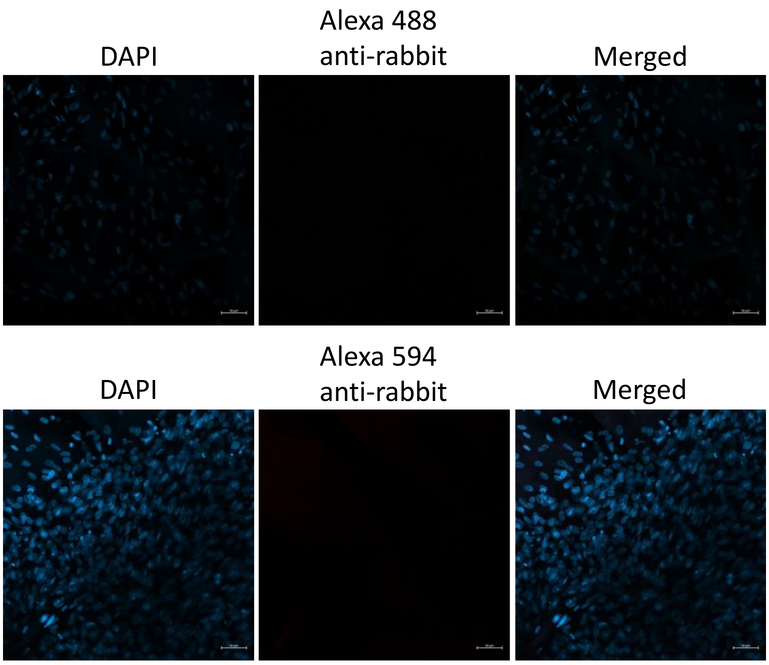

Figure S2 – Immunofluorescence micrograph of ASC under chondrogenic conditions for 7 days stained with Sox9 (Alexa 488 anti-rabbit) and ACAN (Alexa 594 anti-rabbit) secondary antibodies. Scale bar: 50 µm.

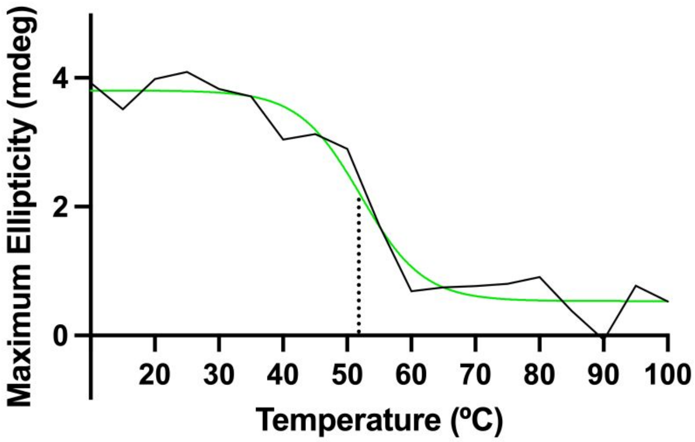

Figure S3 – Variation of the maximum ellipticity of C. reniformis collagen CD spectrum at a fixed wavelength (225 nm) in relation to temperature. The Boltzmann sigmoid function was used to fit a trend line through the experimental data, generating a sigmoidal curve (green line), from which the inflection point was calculated (dotted line). The inflection point (52.1 °C) corresponds to the average transition temperature at which C. reniformis collagen undergoes its structural transition, marking the onset of denaturation.

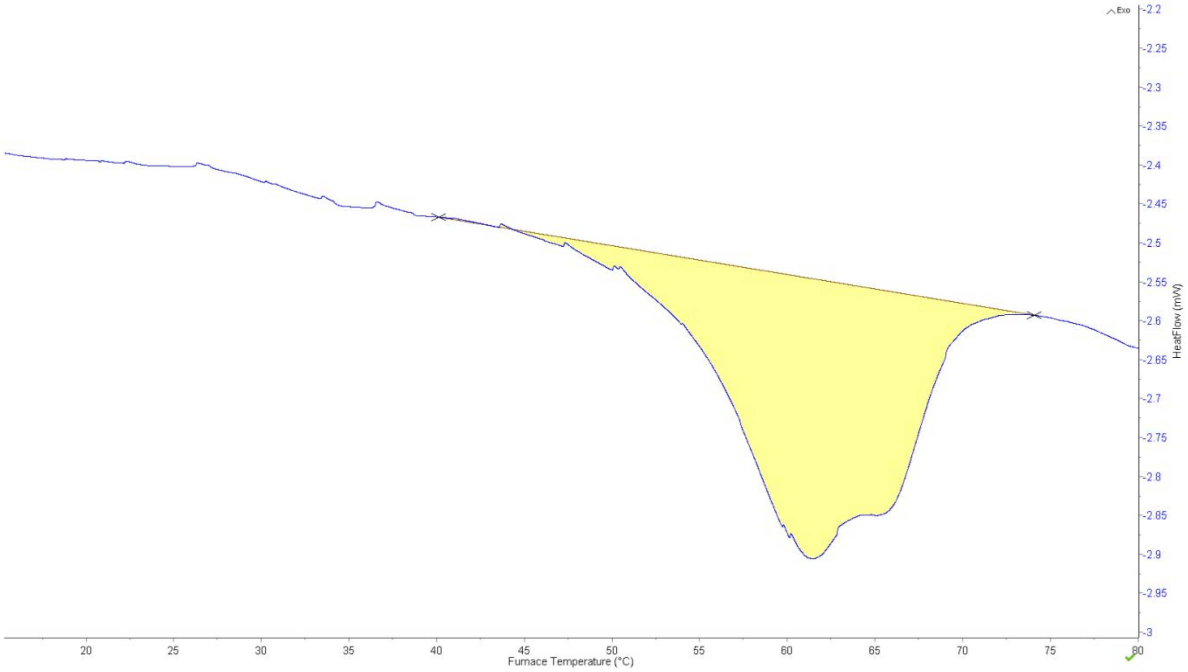

Figure S4 – Representative graph of the thermal analysis of C. reniformis *collagen performed in the microcalorimeter. The endothermic peak representative of collagen denaturation is highlighted in yellow.*
